# Supplementary material for: Baseline Characterization of the Gut Microbiota of Field and Colony Populations of Phlebotomus tobbi and Preliminary Assessment of the Anti-Leishmanial Activity of Cultivable Bacteria
Source: Pathogens. 2026 Jun 23;15(7):658. doi: 10.3390/pathogens15070658 (PMC13416292; doi:10.3390/pathogens15070658)
Supplement: Supplementary file 1 [file pathogens-15-00658-s001.zip › pathogens-4357168-File S1/Taxa2ASV_Decomposer/Genus/g__Serratia/seqs.align.html]

```
seq24           AGTGGGGAATATTGCACAATGGGCGCAAGCCTGATGCAGCCATGCCGCGTGTGTGAAGAA
seq27           AGTGGGGAATATTGCACAATGGGCGCAAGCCTGATGCAGCCATGCCGCGTGTGTGAAGAA
seq61           AGTGGGGAATATTGCACAATGGGCGCAAGCCTGATGCAGCCATGCCGCGTGTGTGAAGAA
seq3            AGTGGGGAATATTGCACAATGGGCGCAAGCCTGATGCAGCCATGCCGCGTGTGTGAAGAA
seq14           AGTGGGGAATATTGCACAATGGGCGCAAGCCTGATGCAGCCATGCCGCGTGTGTGAAGAA

seq24           GGCCTTCGGGTTGTAAAGCACTTTCAGCGAGGAGGAAGGgtagtgtgTTAATAgcAcatT
seq27           GGCCTTCGGGTTGTAAAGCACTTTCAGCGAGGAGGAAGGgttcagtgTTAATAgcActgT
seq61           GGCCTTCGGGTTGTAAAGCACTTTCAGCGAGGAGGAAGGTGGTGAGCTTAATACGcTCAT
seq3            GGCCTTCGGGTTGTAAAGCACTTTCAGCGAGGAGGAAGGTGGTGAGCTTAATACGcTCAT
seq14           GGCCTTCGGGTTGTAAAGCACTTTCAGCGAGGAGGAAGGTGGTGAaCTTAATACGtTCAT

seq24           gcATTGACGTTACTCGCAGAAGAAGCACCGGCTAACTCCGTGCCAGCAGCCGCGGTAATA
seq27           tcATTGACGTTACTCGCAGAAGAAGCACCGGCTAACTCCGTGCCAGCAGCCGCGGTAATA
seq61           CAATTGACGTTACTCGCAGAAGAAGCACCGGCTAACTCCGTGCCAGCAGCCGCGGTAATA
seq3            CAATTGACGTTACTCGCAGAAGAAGCACCGGCTAACTCCGTGCCAGCAGCCGCGGTAATA
seq14           CAATTGACGTTACTCGCAGAAGAAGCACCGGCTAACTCCGTGCCAGCAGCCGCGGTAATA

seq24           CGGAGGGTGCAAGCGTTAATCGGAATTACTGGGCGTAAAGCGCACGCAGGCGGTTTGTTA
seq27           CGGAGGGTGCAAGCGTTAATCGGAATTACTGGGCGTAAAGCGCACGCAGGCGGTTTGTTA
seq61           CGGAGGGTGCAAGCGTTAATCGGAATTACTGGGCGTAAAGCGCACGCAGGCGGTcTGTcA
seq3            CGGAGGGTGCAAGCGTTAATCGGAATTACTGGGCGTAAAGCGCACGCAGGCGGTTTGTTA
seq14           CGGAGGGTGCAAGCGTTAATCGGAATTACTGGGCGTAAAGCGCACGCAGGCGGTTTGTTA

seq24           AGTCAGATGTGAAATCCCCGcGCTtAACgTGGGAACTGCATTTGAAACTGGCAAGCTAGA
seq27           AGTCAGATGTGAAATCCCCGcGCTtAACgTGGGAACTGCATTTGAAACTGGCAAGCTAGA
seq61           AGTCgGATGTGAAATCCCCGGGCTCAACCTGGGAACTGCATTcGAAACTGGCAgGCTAGA
seq3            AGTCAGATGTGAAATCCCCGGGCTCAACCTGGGAACTGCATTTGAAACTGGCAAGCTAGA
seq14           AGTCAGATGTGAAATCCCCGGGCTCAACCTGGGAACTGCATTTGAAACTGGCAAGCTAGA

seq24           GTCTTGTAGAGGGGGGTAGAATTCCAGGTGTAGCGGTGAAATGCGTAGAGATCTGGAGGA
seq27           GTCTTGTAGAGGGGGGTAGAATTCCAGGTGTAGCGGTGAAATGCGTAGAGATCTGGAGGA
seq61           GTCTTGTAGAGGGGGGTAGAATTCCAGGTGTAGCGGTGAAATGCGTAGAGATCTGGAGGA
seq3            GTCTcGTAGAGGGGGGTAGAATTCCAGGTGTAGCGGTGAAATGCGTAGAGATCTGGAGGA
seq14           GTCTcGTAGAGGGGGGTAGAATTCCAGGTGTAGCGGTGAAATGCGTAGAGATCTGGAGGA

seq24           ATACCGGTGGCGAAGGCGGCCCCCTGGACAAAGACTGACGCTCAGGTGCGAAAGCGTGGG
seq27           ATACCGGTGGCGAAGGCGGCCCCCTGGACAAAGACTGACGCTCAGGTGCGAAAGCGTGGG
seq61           ATACCGGTGGCGAAGGCGGCCCCCTGGACAAAGACTGACGCTCAGGTGCGAAAGCGTGGG
seq3            ATACCGGTGGCGAAGGCGGCCCCCTGGACgAAGACTGACGCTCAGGTGCGAAAGCGTGGG
seq14           ATACCGGTGGCGAAGGCGGCCCCCTGGACgAAGACTGACGCTCAGGTGCGAAAGCGTGGG

seq24           GAGCAAA
seq27           GAGCAAA
seq61           GAGCAAA
seq3            GAGCAAA
seq14           GAGCAAA
```
